# Tangluoning, a traditional Chinese medicine, attenuates *in vivo* and *in vitro* diabetic peripheral neuropathy through modulation of PERK/Nrf2 pathway

**DOI:** 10.1038/s41598-017-00936-9

**Published:** 2017-04-21

**Authors:** Xinwei Yang, Weijie Yao, Haolong Liu, Yanbin Gao, Renhui Liu, Liping Xu

**Affiliations:** School of Traditional Chinese Medicine, Capital Medical University and Beijing Key Lab of TCM Collateral Disease Theory Research, Beijing, China

## Abstract

Prolonged hyperglycemia-induced oxidative stress and endoplasmic reticulum stress have been demonstrated to play a key role in progression of diabetic peripheral neuropathy (DPN). PERK/ Nrf2 pathway plays a predominant role in oxidative and endoplasmic reticulum (ER) stress which is associated with cell survival. This study examined the modulation of the PERK/Nrf2 pathway and apoptosis by a traditional Chinese medicine Tangluoning (TLN) in streptozotocin-induced DPN rat models and the effects of serum TLN on the PERK/Nrf2 pathway, apoptosis, intracellular reactive oxygen species and mitochondrial membrane potential in Schwann cells cultured in 150 mM glucose. It is found that TLN attenuated oxidative and ER stress and apoptosis through the PERK/Nrf2 pathway by upregulating p-PERK, Nrf2/ARE pathways and downregulating the CHOP-related apoptosis pathways in the experimental DPN models both *in vivo* and *in vitro*.

## Introduction

Diabetic peripheral neuropathy (DPN) is characterized by high morbidity and premature mortality. It occurs in approximately 50% of diabetic patients^1, 2^. The pathogenesis of DPN results in a widespread damage to all components of peripheral nervous system (PNS) such as dorsal rout ganglia, neurons, vasa nervorum, and primarily, the Schwann cells^3^. Chronic hyperglycemia induces both oxidative stress and endoplasmic reticulum (ER) stress, which are two key factors leading to neuronal apoptosis^4, 5^, and then contributing to DPN^6, 7^.

Oxidative stress induces the accumulation of unfolded and misfolded proteins in the ER lumen, and subsequently unfolded protein response (UPR) by activating specialized sensors including inositol-requiring 1α (IRE1α), RNA-dependent protein kinase (PKR)-like ER kinase (PERK) and transcription factor 6 (ATF6)^8, 9^. When UPR fails to clear misfolded proteins and re-establish protein homeostasis within the ER, pro-apoptotic signaling pathways are activated^4^. Accumulated evidences suggest that the PERK/nuclear factor-E2-related factor 2 (Nrf2) pathways play predominant roles in oxidative and ER stress. PERK-dependent phosphorylation triggers the dissociation of Nrf2/Kelch-like ECH-associated protein 1 (Keap1) complexes and allows subsequent nuclear import of Nrf2, thus activating a set of phase II detoxifying enzymes to combat oxidative and ER stress-induced apoptosis^10–12^.

The most significant ER stress-induced apoptotic pathway is mediated through the C/EBP homologous protein (CHOP)^7^. CHOP can down regulate Bcl-2 while increasing the translocation of Bax from cytosol to mitochondria, which consequently triggers caspase 3-independent apoptosis in both ER and mitochondria^13, 14^. Bcl-2 and Bax are the molecular targets located on the cytoplasmic side of the outer mitochondrial membrane and ER, and are cross-talk between oxidative stress and ER stress^15, 16^. Schwann cells (SCs) are sensitive to high glucose concentration^17^. Hyperglycemia-induced SC damages result in reduced nerve conduction velocity, axonal atrophy, and impaired axonal regeneration, and are a cause for DPN^18, 19^.

Recent *in vivo* studies have reported that ER stress plays a key role in the pathogenesis of peripheral neuropathy in prediabetic and type 1 diabetic rats^20, 21^. However, little attention has been focused on the effect of ER stress on the lesions of SCs in models of DPN. Traditional Chinese medicine Tangluoning (TLN) based on Huangqi Guizhi Wuwu decoction is clinically used to treat DPN in China^22^. Our previous study has shown that TLN markedly improves the neurological functions including thermal perception threshold and nerve conductivity in DPN rats by attenuating oxidative stress through the activation of Nrf2^23^. It is possible that TLN may affect ER stress sensor PERK and attenuate DPN through modulating oxidative and ER stress. The goal of the present study was to investigate whether TLN improves DPN by alleviating both oxidative and ER stress through modulating the PERK/Nrf2 pathway.

## Results

### TLN upregulates PERK/Nrf2 pathway in sciatic nerve of STZ-induced DPN rats

**Figure 1:**
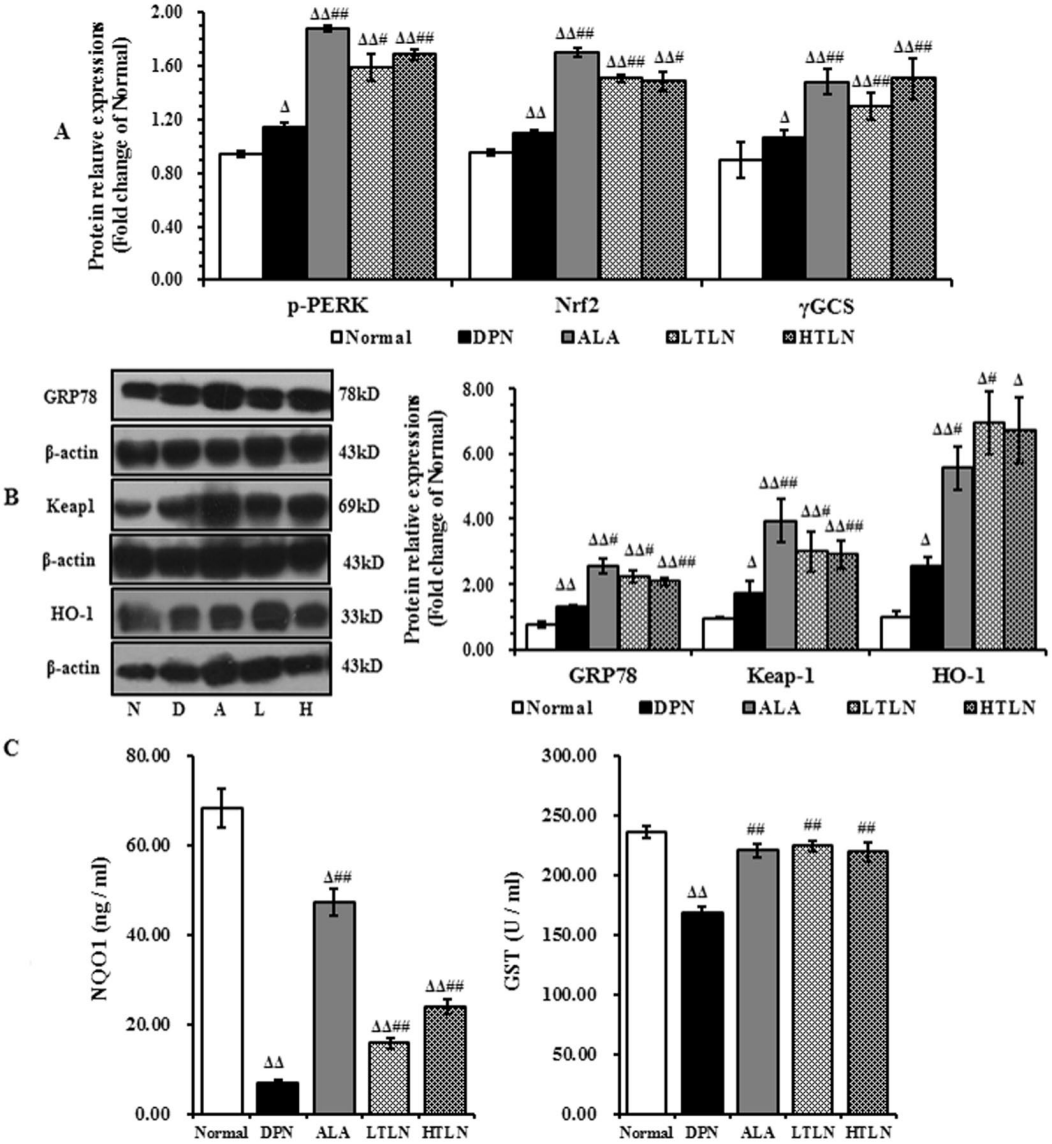
Up-regulation of the PERK/Nrf2 pathway in sciatic nerve of STZ-induced DPN rats by TLN. (**A**) The relative expression of p-PERK, Nrf2 and γGCS determined by double-immunostaining 12 weeks after TLN treatment. (**B**) Representative Western blot of GRP78, Keap1 and HO-1 12 weeks after TLN treatment (Left panel), and the relative fold changes of GRP78, Keap1 and HO-1. (**C**) The serum levels of NQO1 and GST 12 weeks after TLN treatment. NQO1 was detected using the ELISA kit. GST activity was determined using a colorimetric method. Data are shown as mean ± S.E.M. ^Δ^ *P* < 0.05, ^ΔΔ^ *P* < 0.01 vs. Normal group; ^#^ *P* < 0.05, ^##^ *P* < 0.01 vs. DPN group. Data were analyzed by One-way ANOVA followed by least significant difference or Tambane’s T2 analysis (n = 6 animals per group).

Our previous study demonstrated that TLN improves oxidative stress by upregulating the expression of Nrf2 in STZ-induced DPN rats^23^. Nrf2 is a direct PERK substrate, and contributes to cellular redox homeostasis by regulating phase II antioxidant response^24^. Therefore, we examined whether TLN could activate the PERK/Nrf2/oxidative stress signaling pathway in sciatic nerves. An upregulation of the ER chaperone glucose-regulated protein 78 (GRP78) was detected in the DPN rats using western blot analysis compared to normal rats, which was further increased in TLN-treated rats (Fig. 1B). The expression of phosphorylated PERK (p-PERK), Keap1 and Nrf2 was also up-regulated significantly following TLN treatment (Fig. 1A,B and Supplementary Fig. S1). Similarly, a significant upregulation of the PERK/Nrf2-target downstream proteins γGCS, HO-1, GST and NQO1 was observed in TLN-treated rats (Fig. 1A and Supplementary Fig. S1, Fig. 1B,C). These results demonstrate that TLN might play a protective role in oxidative stress in the DPN rats by up-regulating the PERK/Nrf2 signaling pathways.

### TLN inhibits CHOP-induced apoptosis in the sciatic nerve of STZ-induced DPN rats

**Figure 2:**
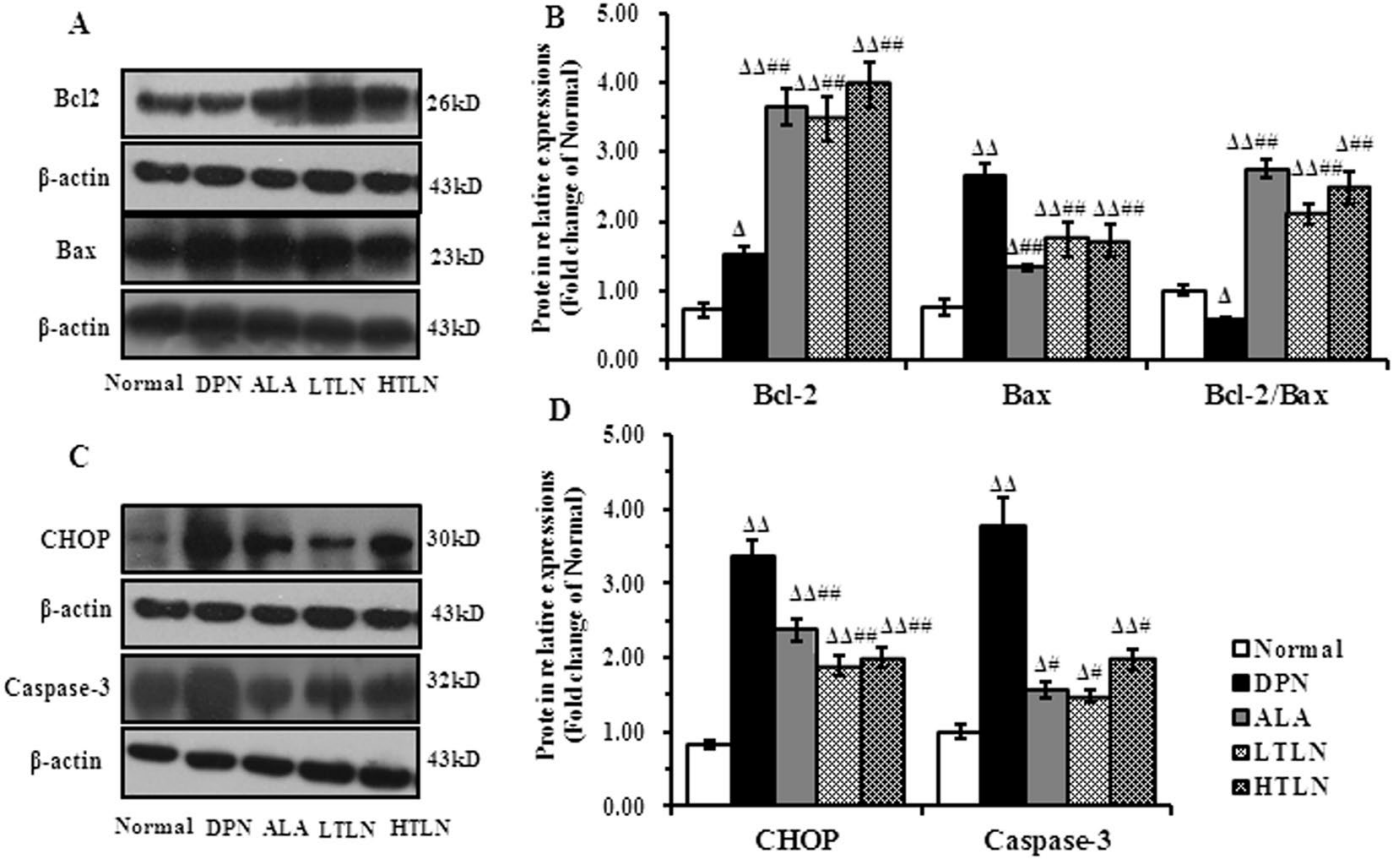
Downregulation of CHOP, Bax, Caspase-3 and upregulation of Bcl-2 in sciatic nerve of STZ-induced DPN rats by TLN treatment. (**A**) and (**C**) Representative Western blots of Bcl-2, Bax, CHOP and Caspase-3 12 weeks after TLN treatment (Left panel). (**B**) and (**D**) The relative levels of Bcl-2, Bax, CHOP and Caspase-3 (Right panel). Data are shown as mean ± S.E.M. ^Δ^ *P* < 0.05, ^ΔΔ^ *P* < 0.01 vs. Normal group; ^#^ *P* < 0.05, ^##^ *P* < 0.01 vs. DPN group. Data were analyzed by One-way ANOVA followed by least significant difference or Tambane’s T2 analysis (n = 6 animals per group).

Oxidative and ER stress-induced apoptosis is a critical event in DPN^12^. This study investigated whether TLN inhibits apoptosis through upregulating PERK/Nrf2 pathway. Apoptosis-related proteins downstream of PERK were observed, such as CHOP, the ER-apoptotic marker, and members of the BCL-2 family. Western blot analysis showed that the expression of CHOP, Bax/Bcl-2 and Caspase-3 in the DPN rats was increased compared with the normal rats, whereas TLN treatment decreased their expression (Fig. 2).

### TLN decreases high glucose-induced apoptosis and oxidative stress in RSC96 cells

**Figure 3:**
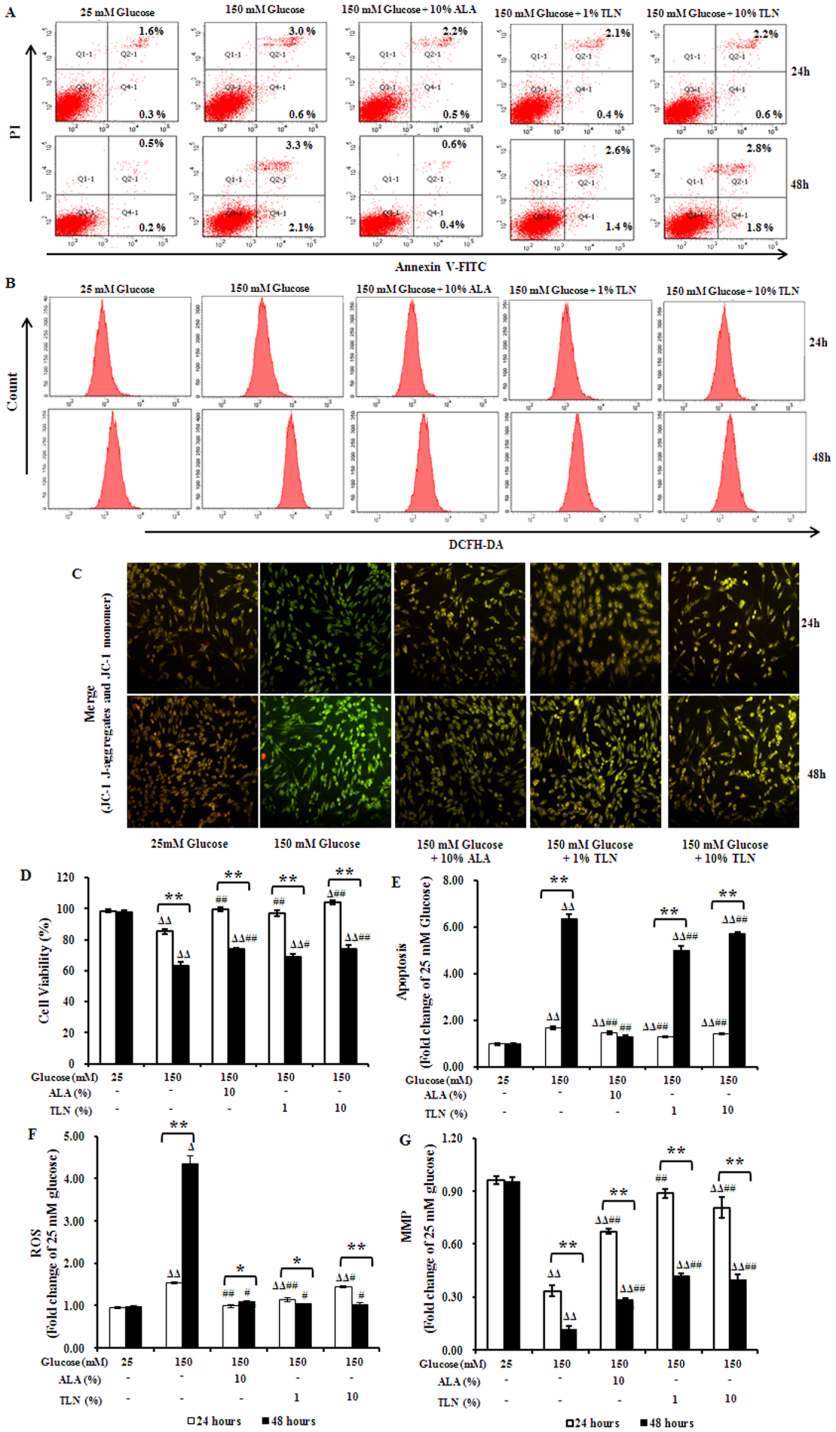
Apoptosis and oxidative stress in RSC96 cells treated with TLN and exposed to high glucose. (**A**) FACS apoptosis assay results using FITC-Annexin V and PI staining at 24 h and 48 h. (**B**) Intracellular ROS production measured with FACS analysis using DCHF-DA as a fluorescent dye at 24 h and 48 h. (**C**) The mitochondrial membrane potential (MMP) detected using JC-1. The decrease in MMP is reflected by a decrease in red fluorescence in the merged images. (**D**) Cell viability determined using MTT assay. **(E–G)** Levels of apoptosis, MMP and ROS shown as fold changes relative to 25 mM glucose-treated cells. Data are represented as the mean ± S.E.M. ^Δ^ *P* < 0.05, ^ΔΔ^ *P* < 0.01 vs. 25 mM glucose groups; ^#^ *P* < 0.05, ^##^ *P* < 0.01 vs. 150 mM glucose groups; ^*^ *P* < 0.05, ^**^ *P* < 0.01 vs. treated after 24 hours. Data were analyzed by One-way ANOVA followed by least significant difference or Tambane’s T2 analysis. Student’s unpaired t-test compared to 24 hours’ time point. (n = 4 per group).

High glucose in the peripheral nervous system (PNS), specifically in the Schwann cell-rich sciatic nerve plays a key role in the pathogenesis of DPN^25, 26^. To further confirm the effects of TLN on DPN, high glucose-treated Schwann cells (SCs) were used for investigation. For this purpose, RSC96 cells were co-treated with high level of glucose (150 mM) and different concentrations of TLN. As shown in Fig. 3D, 86% of the cells survived following 150 mM glucose treatment for 24 hours compared with the 25 mM glucose treatment; 1% and 10% TLN increased the survival to 94% and 103%, respectively. 48 hours after the treatment, 66% of the cells survived following 150 mM glucose treatment compared with the 25 mM glucose treatment, and 72% and 76% of the cell survived when co-treated with 1% and 10% TLN, respectively.

Flow cytometry showed that cells treated with 150 mM glucose had 1.7-fold increase in apoptosis as compared with 25 mM glucose after 24 hours of incubation, while co-treatment with TLN reduced the increase to 1.3–1.4 fold. After 48 hours, 150 mM glucose induced 6.3-fold increase in apoptosis as compared with 25 mM glucose, while co-treatment with 1% and 10% TLN decreased the increase to 5.0 and 5.7-fold, respectively (Fig. 3A,E).

We further studied whether TLN could affect the production of intracellular ROS in RSC96 cells. FACS analysis showed that ROS level was continuously increased following treatment with 150 mM glucose for 24 h and 48 h as compared with 25 mM glucose. However, co-treatment with 1% and 10% TLN continuously diminished the ROS production at 24 h and 48 h (Fig. 3B,F).

To investigate the contribution of PERK/Nrf2 activation to mitochondrial dysfunction and ROS generation, we evaluated the level of MMP in high glucose treated-cells using JC-1. After exposed to 150 mM glucose, more RSC96 cells were depolarized for mitochondrial membrane, showing lower MMP after the exposure at 24 hours and 48 hours. On contrast, co-treatment with 1% and 10% TLN increased the MMP (Fig. 3C,G).

### TLN upregulates the PERK/Nrf2 pathway in RSC96 cells exposed to high glucose

**Figure 4:**
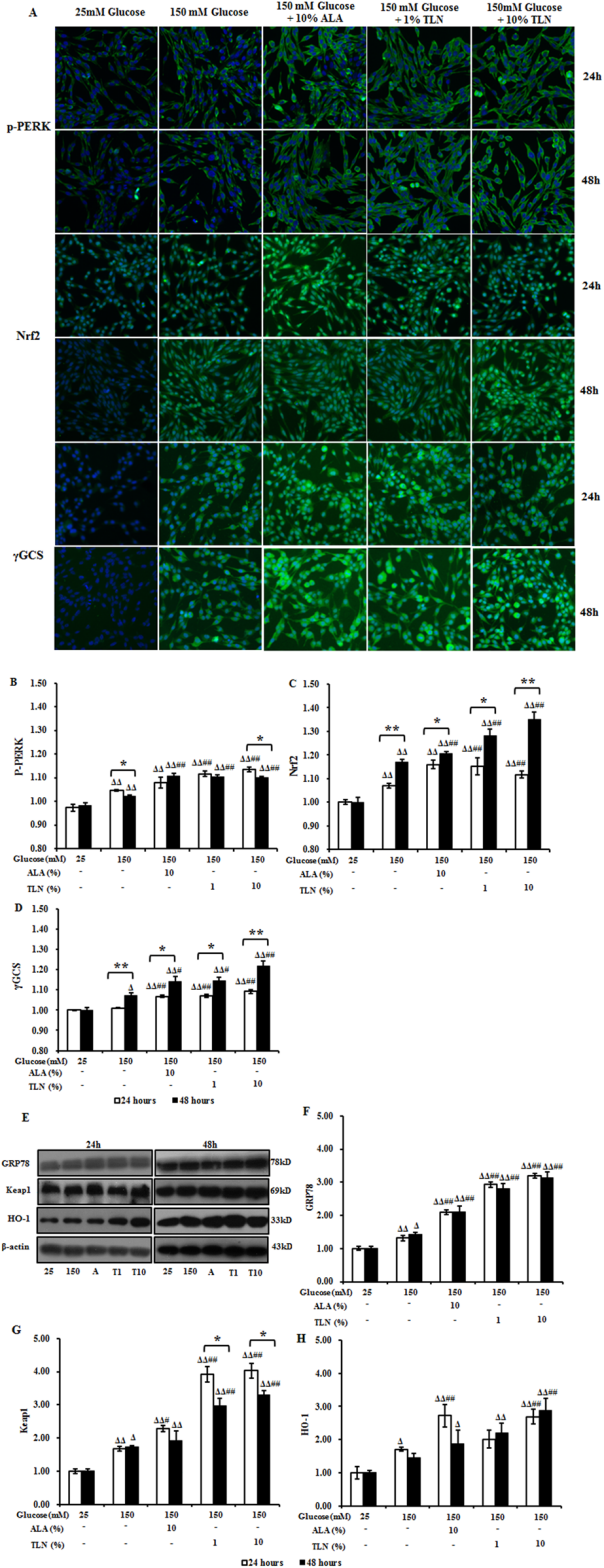
Expression of proteins in the PERK/Nrf2 pathway in RSC96 cells treated with TLN and exposed to high glucose. (**A**) High content analysis of p-PERK, Nrf2 and γGCS at 24 and 48 h. Images show immunostaining of RSC96 cells (10× magnification). (**B**–**D**) Fold changes of p-PERK, Nrf2 and γGCS levels relative to these after 25 mM glucose treatment. (**E**) Representative Western blots of GRP78, Keap1 and HO-1 in RSC96 cells 24 and 48 h. (**F**–**H**) Relative protein levels of GRP78, Keap1 and HO-1 against of β-actin. Data are represented as the means ± S.E.M. ^Δ^ *P* < 0.05, ^ΔΔ^ *P* < 0.01 vs. 25 mM glucose groups; ^#^ *P* < 0.05, ^##^ *P* < 0.01 vs. 150 mM glucose groups; ^*^ *P* < 0.05, ^**^ *P* < 0.01 vs. treated after 24 hours. Data were analyzed by One-way ANOVA followed by least significant difference or Tambane’s T2 analysis and Student’s unpaired t-test compared to 24 hours’ time point. (n = 4 per group).

Recently, mounting evidence has revealed that the PERK/Nrf2 pathway plays a key role in mitochondrial dysfunction and ROS generation^27^. Therefore, we analyzed whether or not TLN was responsible for attenuating the mitochondrial dysfunction and ROS generation in RSC96 cells after exposed to high-glucose through the PERK/Nrf2 pathway. The exposure up-regulated the expression of GRP78, p-PERK, Keap1, Nrf2 and HO-1 24 and 48 hours after the treatment as compared with the untreated cells, and no significant change in γGCS expression was observed at 24 h. Furthermore, the expression of p-PERK, Keap1, Nrf2, HO-1 and γGCS was up-regulated in co-treatment with TLN at 24 and 48 h. On other hand, the protein levels of phosphorylated PERK and Keap1were gradually decreased, while the levels of Nrf2, HO-1 and γGCS were increased at 24 and 48 h (Fig. 4).

### TLN inhibites CHOP induced-apoptosis in RSC96 cells exposed to high glucose

**Figure 5:**
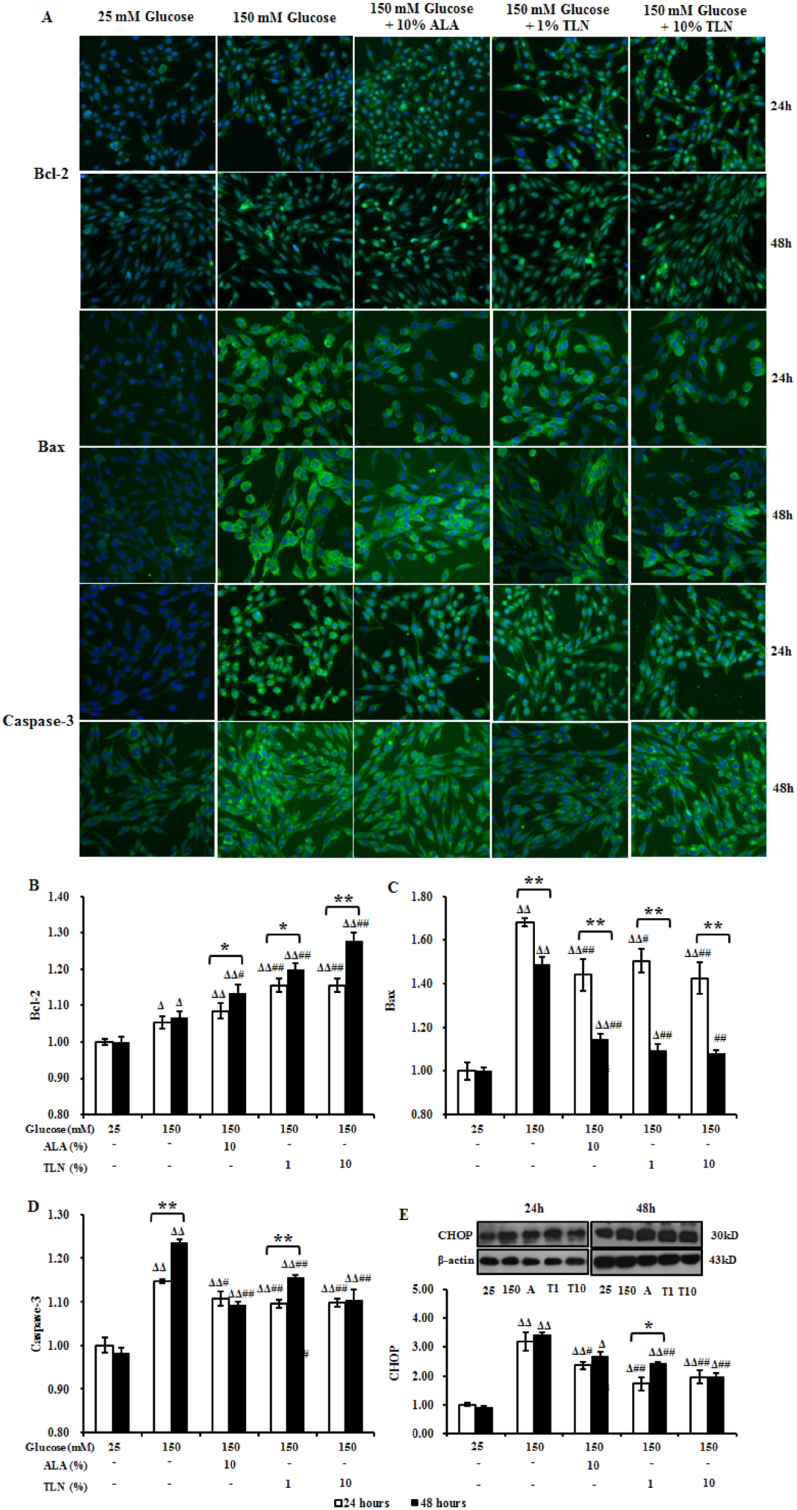
Effects of TLN on the expression of CHOP, Bcl-2, Bax and Caspase-3 in RSC96 Cells. (**A**) High content analysis of Bcl-2, Bax and Caspase-3 at 24 and 48 h. Images show immunostaining of RSC96 cells (10× magnification). (**B**–**D)** Fold changes of Bcl-2, Bax and Caspase-3 relative to these in 25 mM glucose-treated cells. (**E**) Western blots of CHOP in RSC96 cells at 24 and 48 h and fold changes relative to these in 25 mM glucose-treated cells. Data are represented as the means ± S.E.M. ^Δ^ *P* < 0.05, ^ΔΔ^ *P* < 0.01 vs. 25 mM glucose groups; ^#^ *P* < 0.05, ^##^ *P* < 0.01 vs. 150 mM glucose groups; ^*^ *P* < 0.05, ^**^ *P* < 0.01 vs. treated after 24 hours. Data were analyzed by One-way ANOVA followed by least significant difference or Tambane’s T2 analysis and Student’s unpaired t-test compared to 24 hours’ time point. (n = 4 per group).

To characterize the apoptosis exposed to high glucose, we monitored the expression of CHOP. Western analyses showed that after exposed to high glucose for 24 hours and 48 hours, the expression of CHOP was up-regulated and co-treatment with TLN persistently decreased the expression (Fig. 5E). To define whether CHOP inhibits the expression of Bcl-2 while increasing the translocation of Bax from cytosol to mitochondria, which consequently triggers caspase 3-independent apoptosis in both ER and mitochondria, high content analysis was used to analyze the levels of Bcl-2, Bax and caspase-3. We found that caspase-3 and Bax were up-regulated as compared with untreated cells, whereas co-treatment with TLN decreased the expression of Bax and Caspase-3 (Fig. 5A,C,D) and further increased the level of Bcl-2 (Fig. 5A,B). Taken together with our previous results, these observations suggest that CHOP is specifically activated after exposed to high glucose and cold induce apoptosis.

## Discussion

Chronic hyperglycemia is able to initiate both oxidative stress and ER stress, which are two key factors contributing to DPN^7^. Oxidative stress, commonly linked to mitochondrial dysfunction and ER stress has been identified as an important mechanism leading to neuronal apoptosis^28^. PERK/Nrf2 is a cross-talk pathway between oxidative stress and ER stress and plays a key role in cell survival^5, 7^. Therefore, it is likely that simultaneous targeting of oxidative stress, ER stress and apoptosis through the PERK/Nrf2 pathway might produce a better therapeutic response than targeting them individually.

SCs are the most abundant glial cells in the PNS. SC injury is expected to reduce motor and sensory nerve conductivity, impair thermal nociception^29^. This study demonstrates that SCs exposed to high glucose, *in vivo* or *in vitro*, display the classic morphologic features of apoptosis. Apoptosis in SCs is a common feature of human and animal DPN^30^. Moreover, SCs play an extensive role in the regulation of axonal regeneration, hyperglycemia-induced SCs damages may cause reduced nerve conductivity, axonal atrophy, and impaired axonal regeneration^31^. In our previous study, TLN was found to improve the deficiency of motor nerve conductivity, sensory nerve conductivity and thresholds of thermal perception which were accompanied by lesser demyelination and a milder axonal atrophy in DPN rats^23^. It was also found to induce apoptosis in hyperglycemic media^32^. Based on these previous studies, we endeavored to investigate the effect of TLN on oxidative stress and ER stress in DPN rats and high glucose treated-SCs.

Prolonged hyperglycemia is shown to lead to ROS accumulation in mitochondria and ER, and induce oxidative and ER stress^4^. It is implicated as a major risk factor in the onset and progression of DPN^33^. In the present study, two peak levels of ROS following exposure to high glucose for 24 and 48 hours were found, and co-treatment with TLN reduced the ROS accumulation, particularly 48 h after the exposure. Hyperglycemia is associated with increased ROS in oxidative stress and ER stress. ER stress-induced ROS production may also elevate the level of mitochondrial ROS above the threshold for cell survival, leading to cell death. It is proposed that mitochondrial ROS impairs ER function via the enhancement of ER stress^34^. Previously research showed that TLN reduces the level of ROS and apoptosis under hyperglycemic conditions 12 weeks after the treatment in STZ-induced DPN rats but not the hyperglycemia levels. Thus TLN may directly inhibit oxidative stress and ER stress. To confirm the ER stress, GRP78, the key ER regulatory protein which plays an important role in the ER stress, was measured^35^. Under normal conditions, GRP78 is expressed at a low level and bound to ER transmembrane receptors. When ER stress is triggered, the unfolded or misfolded proteins will bind with free GRP78, resulting in its activation^36^. Our results showed the expression of GRP78 was up-regulated 12 weeks after rats were treated with STZ and TLN further increased the expression, suggesting that ER stress is triggered in the DPN rats. TLN induced similar effects on GRP78 expression in a time-dependent manner in SCs exposed to high glucose, indicating that high glucose may cause ER stress. We, therefore, conclude that TLN could activate ER stress-dependent downstream pathways which are essential for survival.

PERK-dependent activation of Nrf2 is required for survival^37^. PERK has been proposed as an Nrf2 activator in the context of ER stress and oxidative stress^24^. During the UPR and oxidative stress, PERK signaling activates Nrf2, the PERK substrate, to dissociate from its negative regulator Keap1 to induce the expression of a set of phase II detoxifying enzymes including hemeoxygenase 1 (HO-1), γ-glutamylcysteine synthetase (γ-GCS), glutathione S-transferase (GST) and NAD(P)H: quinone oxidoreductase 1 (NQO1)^38^. Keap1/Nrf2 dependent antioxidant protection system is considered one of the most important cellular defence mechanisms in scavenging ROS to combat oxidative stress and ER stress^39, 40^. Here, we show that hyperglycemia-induced oxidative stress and ER stress increased the expression of p-PERK in STZ-induced DPN rats, promoted the dissociation of Nrf2 from Keap1 to induce the expression of HO-1, γGCS, GST and NQO1. TLN further activated the PERK/Nrf2 pathway in DPN rat’s sciatic nerve. Similar to vivo studies, *in vitro* studies treated with SCs under hyperglycemic conditions mainly increased the expression of PERK/Nrf2 pathway-related proteins at 24 and 48 hours. These findings further confirm that TLN is effective on oxidative stress and ER stress in DPN.

Apoptosis induced by oxidative and ER stress after hyperglycemia also plays a substantial role in DPN^41^.

CHOP, which is also activated via PERK, plays a crucial role in oxidative and ER stress-induced apoptosis^42^. PERK/Nrf2/oxidative stress signaling has been proven to prevent cells apoptosis^24^. For example, GRP78 and Nrf2 targeting proteins including HO-1 have been suggested to be anti-apoptotic by inhibiting CHOP expression^43, 44^. Anti-apoptotic Bcl-2 and pro-apoptotic Bax have been suggested to regulate the homeostasis in mitochondria in ER stress-initiated apoptosis^45^. CHOP not only down regulates Bcl-2 expression but also increases the translocation of Bax from cytosol to mitochondria, which consequently triggers the activation of caspase-3, resulting in apoptosis^41^. The killer caspases-3 is critical in both the mitochondrial apoptotic pathway and ER stress-induced apoptosis^46^. In our experiment, we found that the expression of CHOP and Caspase-3 and Bax/Bcl-2 ratio were significant increased in sciatic nerve of DPN rats and SCs exposed to high glucose.

An increase of ROS can trigger the opening of the mitochondrial permeable transition pore, which in turn leads to the simultaneous collapse of MMP and a massive release of ROS, resulting in apoptosis. Emerging evidence suggests that prolonged oxidative and ER stress exacerbate mitochondrial dysfunction^47^. Consistent with this, SCs under ER stress induced by high glucose for 24 and 48 h were accompanied by a decrease in MPP. In addition, Annexin V-FITC and PI staining also revealed high glucose-induced apoptosis in SCs. PERK/Nrf2 plays an important role in mitochondrial dysfunction. We found that TLN decreased the Annexin V-FITC and PI stained-cells and inhibited the increase in MPP in SCs. TLN decreased the expression of CHOP, Caspase-3 and Bax/Bcl-2 ratio, increased the MMP, indicating that it could attenuate oxidative and ER stress-induced apoptosis.

**Figure 6:**
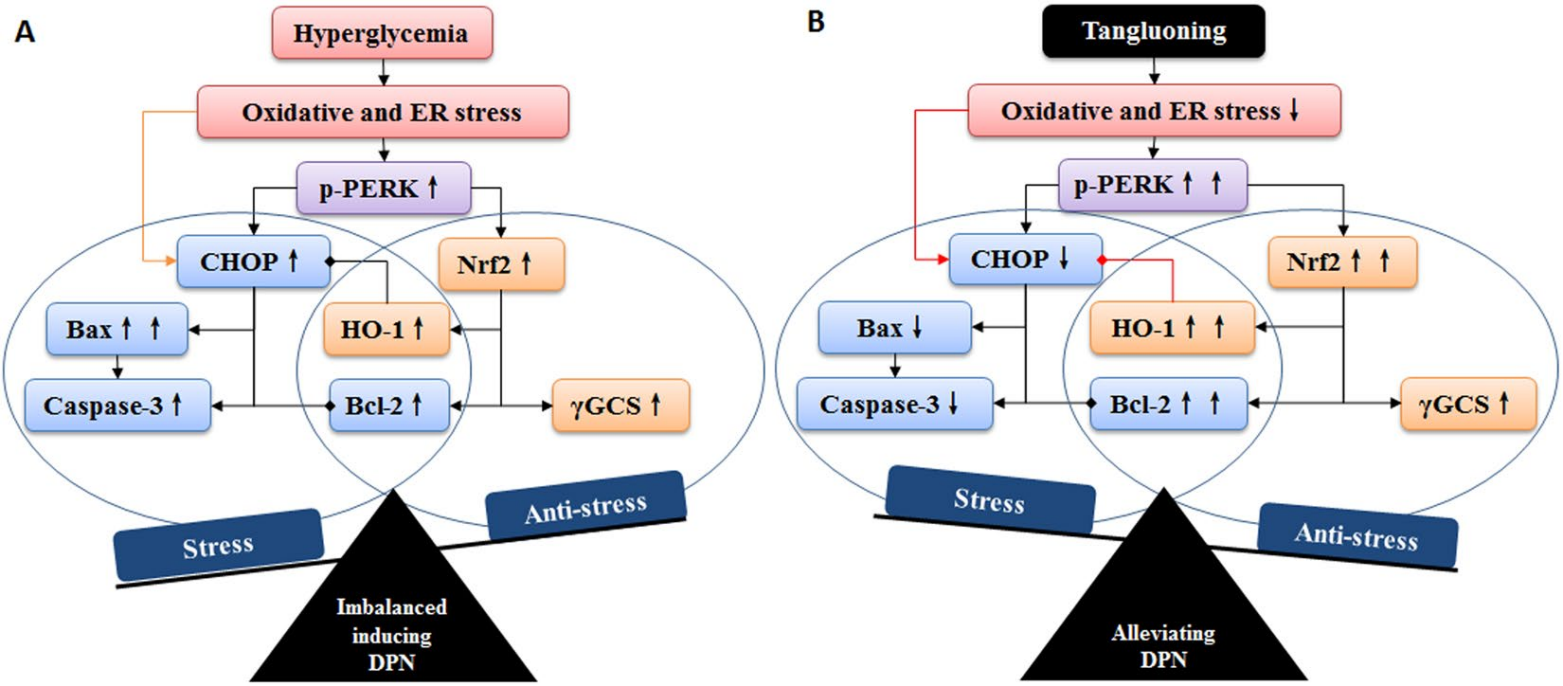
PERK/Nrf2 pathway in diabetic peripheral neuropathy and its regulation by Tangluoning.

According to these results, we hypothesize that hyperglycemia induces oxidative stress and ER stress, and then activates the PERK/Nrf2 pathway and upregulates the expression of CHOP. *In vivo* or *in vitro* DPN model, increased p-PERK can both activate the Keap1/Nrf2 pathway and CHOP-induced apoptosis pathway. The results of apoptosis assay and intracellular ROS analysis show that high glucose increases the expression of p-PERK but does not alleviate oxidative stress. There may be other mechanisms that upregulate CHOP-induced apoptosis and break the balance between Keap1/Nrf2 antioxidative pathway and CHOP-induced apoptosis pathway, resulting in DPN. Tangluoning reduces the oxidative stress and ER stress, and functions as an activator to further activate the PERK/Nrf2 pathway and upregulate the expression of antioxidant enzymes including HO-1 and γGCS. HO-1 has been reported to inhibit the expression of CHOP while Keap1/Nrf2 upregulates the expression of Bcl-2. Tangluoning decreased the Bax/Bcl-2 ration, thus inhibiting apoptosis, oxidative stress and ER stress and improving DPN (Fig. 6). Further work will be necessary to fully define the potential target of the PERK/Nrf2 pathway in DPN progression.

## Materials and Methods

### TLN Preparation

The formula of TLN per dose is listed in Suppl. Table 1. Eleven compounds (Suppl. Fig. 2) of TLN were characterized using HPLC–MS/MS and five compounds were quantified (Suppl. Table. 2). Briefly, the following crude herbs were combined to make the TLN extract powder: 15.0 g of Huangqi, 15.0 g of Danshen, 15.0 g of Jixueteng, 15.0 g of Mugua, 15.0 g of Gouji, 12.0 g of Chishao, 12.0 g of Niuxi and 10.0 g of Yanhusuo. The protocol of preparation TLN powder was as previously described^23^ and the powder was dissolved in distilled water.

### Treatment of diabetic rats with TLN

The experimental procedures were carried out following the guidelines of the Experimental Animal Care and Use Committee at the Capital Medical University (Beijing, China). All studies were approved by the Ethics Review Committee for Animal Experimentation of Capital Medical University (Ethical Approval Number 2013-X-42). Male Sprague-Dawley (SD) rats (weighing 200 ± 20 g) were obtained from the Experimental Animal Center at Capital Medical University, Beijing, China (SCXK 2012–0001) and were housed on a 12 hours on and 12 hours off light cycle at 23 ± 2 °C in rooms with 55 ± 10% relative humidity and allowed free access to drinking water and standard laboratory chows. Experimental design, protocols and sample collections were the same as previously described^23^. In brief, rats were randomly divided into normal, DPN, α-lipoic acid (ALA), low dose TLN (LTLN) and high dose TLN (HTLN) groups. Rats were fasted for 12 hours and intraperitoneal injected with STZ (60 mg/kg, dissolved in 0.1 M citrate buffer, pH 4.5 at 4 °C), except for normal group rats. After 72 hours, rats with fasting blood glucose (FBG) concentration greater than or equal to 16.7 mM were considered diabetes and were used for further experiments. ALA, LTLN and HTLN group rats were intragastrically administered with ALA suspension (20 mg/kg/day), TLN extract 10.9 g crude drug/kg/day and 21.8 g crude drug/kg/day, respectively for 12 weeks. In order to prevent the death of rats before the experimental endpoint due to high blood glucose, rats with FBG greater than 25.5 mM were subcutaneously injected with isophane protamine and biosynthetic human insulin.

Rats in ALA, LTLN and HTLN groups were intragastrically administered with ALA suspension (20 mg/kg/day), TLN extract 10.9 g crude drug/kg/day and 21.8 g crude drug/kg/day, respectively, and rats in normal and DPN groups rats were given an equal volume of distilled water everyday day for 12 weeks.

### Serum preparation

30 SD rats were divided into normal (n = 15), ALA (n = 5), and TLN (n = 10) gropus, and were intragastrically administered with distilled water, 20 mg/kg/day ALA suspension and 10.9 g crude drug/kg/day TLN respectively for 8 days. One hour after the last administration, rats were anesthetized with 10% chloral hydrate (i.p., 0.35 g/kg body weight). Blood was sterilely collected through the ventral aorta. After settling for 2 h at room temperature, the blood samples were centrifuged at 3000 r/min at 4 °C for 15 min, and inactivated at 56 °C for 30 min. The samples were stored at −80 °C after filtered through a microfiltrate membrane (0.22 μm).

### Treatment of RSC96 cells cultured with 150 mM glucose and TLN

RSC96 cells (CRL-2765) were purchased from the American Type Culture Collection (ATCC) and were cultured in DMEM modified to contain 4 mmol L-glutamine, 25 mM glucose, 1 mmol sodium pyruvate, 1500 mg/L sodium bicarbonate and 10% FBS at 37 °C in a humidified atmosphere of 5% CO_2_. Cells were passaged once every 3 days. Cells were allowed to adhere for 24 h, and then treated with 25 mM glucose (containing 10% normal rat serum), 150 mM glucose (containing 10% normal serum), 150 mM glucose +10% ALA serum, 150 mM glucose +0.1% TLN serum (containing 0.1% TLN serum +9.9% normal serum), 150 mM glucose +1% TLN serum (containing 1% TLN serum +9% normal serum) and 150 mM glucose +10% TLN serum for 24 and 48 hours, respectively.

### Measurement of NQO1 and GST

After removal of the sciatic nerve, blood samples were collected, and centrifuged at 3000 rpm for 10 min at 4 °C to obtain serum. All serum samples were stored at −80 °C. NQO1 was detected using the commercial enzyme-linked immunosorbent assay (ELISA) kits (Bluegene Biotech, Shanghai, China). GST activity was determined by using a kit (Nanjing Jiancheng Bioengineering Institute, Nanjing, China). All experimental procedures were performed according to the manufacturer’s instructions.

### Double-immunostaining for Rat Sciatic Nerve

At the end of the 12th week, rats were anesthetized with 10% chloral hydrate (i.p., 0.35 g/kg body weight). The left sciatic nerve (1 cm) was fixed in 10% buffered formalin and processed for paraffin section. 4 μm thick transverse sections were regularly dewaxed, repaired antigen retrieval with hot citric acid buffer (pH 6.0) and blocked with 3% BSA. The sections were incubated with primary antibodies against p-PERK, Nrf2, γGCS, respectively and S100β (staining for Schwann cells) at room temperature for 2 hours, followed by incubation with FITC-conjugated goat anti-rabbit IgG (1:100) and FRITC-conjugated goat anti-mouse IgG (1:200) (Zhong Shan Golden Bridge Biotechnology, Beijing, China). Antibodies used are listed in Suppl. Table 3.

### Western Blot

Sciatic nerves and RSC96 cells were lysed on ice in the RIPA buffer with protease inhibitor cocktail for 15 min to extract total proteins. The proteins were analyzed with a bicinchoninic acid (BCA) protein assay kit and 30 μg/lane (sciatic nerve) and 20 μg/lane (RSC96 cell) were used for Western blot analysis as previously described^23^, using antibodies listed in Suppl. Table 3.

### MTT viability assay

The MTT 3-(4,5-dimethyl-2-thiazolyl)-2,5-diphenyl- tetrazolium bromide was purchased from Sigma-Aldrich, USA. Assay analysis was performed as previously described^32^. Briefly, 20 μL of MTT solution (5 mg/mL) was added and incubated for 4 h at 37 °C. The supernatant was removed and 150 μL of DMSO/well was added to dissolve the intracellular crystalline formazan product. Cell viability was determined by measuring the absorbance at a wavelength of 490 nm.

### Apoptosis Assay

Apoptosis was detected using an Annexin-FITC Apoptosis Detection Kit (No. AP101, Multisciences, China). Cells were collected and washed with PBS. After the addition of 500 μL of binding buffer, 5 μL of FITC-labeled annexin V and 10 μL of propidium iodide (PI) were added and incubated at room temperature for 5 min in the dark. Stained cells were analyzed using BD LSRFortessa™ flow cytometer (BD Biosciences, San Jose, CA, USA).

### Measurement of Mitochondrial Membrane Potential (MMP)

MMP of cells was measured using a mitochondrial membrane potential assay kit (Cat no. C2006, Beyotime, China). In brief, cells were stained with JC-1 solution (500 μL/well) and incubated at 37 °C for 20 min in the dark. A fluorescence microscope was used to check the fluorescence. Polarized J-aggregated mitochondria and depolarized monomer mitochondria were analyzed through TRITC channel and FITC channel, respectively. MMP was expressed as the ratio of JC-1 J-aggregates IOD/JC-1 monomer IOD.

### Intracellular ROS Analysis

ROS in RSC96 cells was detected using a total ROS assay kit (Cat no. 88-5930, eBioscience, USA) via the DCFH-DA method according to the manufacturer’s instructions. Cells were collected and incubated in 1 mL serum-free DMEM containing 100 μL ROS Assay Stain Solution at 37 °C for 30 min in the dark. The mean fluorescence intensity was analyzed using BD LSRFortessa™ flow cytometry.

### High Content Analysis for RSC96 Cell

Cells were fixed with 4% paraformaldehyde at room temperature. For staining, the cells were rinsed in PBS, followed by permeabilization for 30 min in 0.5% Triton-X 100 and blocked by 3% BSA. Then, the RSC96 cells in 96-well plate was incubated with primary antibodies (Suppl. Table 3) at 4 °C overnight. After washing, cells were incubated with FITC-conjugated goat anti-rabbit IgG (1:200 dilution) at room temperature for 1 h and counterstained with DAPI for 5 min^32, 48^.

### Statistical Analysis

Data were expressed as the means ± S.E.M. Differences were analyzed by Student’s unpaired t-test or One-way ANOVA followed by least significant difference or Tambane’s T2 analysis using SPSS 17.0. *P* < 0.05 were considered to be statistically significant.

## Electronic supplementary material

Dataset 1

## References

[1] Zochodne DW (2007). Diabetes mellitus and the peripheral nervous system: manifestations and mechanisms. Muscle Nerve..

[2] Tian R (2016). Rutin ameliorates diabetic neuropathy by lowering plasma glucose and decreasing oxidative stress via Nrf2 signaling pathway in rats. Eur J Pharmacol..

[3] Chan L, Terashima T, Urabe H, Lin F, Kojima H (2011). Pathogenesis of diabetic neuropathy: bad to the bone. Ann N Y Acad Sci..

[4] Cao SS, Kaufman RJ (2014). Endoplasmic reticulum stress and oxidative stress in cell fate decision and human disease. Antioxid Redox Signal..

[5] Malhotra JD, Kaufman RJ (2007). Endoplasmic reticulum stress and oxidative stress: a vicious cycle or a double-edged sword?. Antioxid Redox Signal..

[6] Feldman EL (2003). Oxidative stress and diabetic neuropathy: a new understanding of an old problem. J Clin Invest..

[7] O’Brien PD, Hinder LM, Sakowski SA, Feldman EL (2014). ER stress in diabetic peripheral neuropathy: A new therapeutic target. Antioxid Redox Signal..

[8] Oñate M (2016). Activation of the unfolded protein response promotes axonal regeneration after peripheral nerve injury. Sci Rep..

[9] Delaunay-Moisan A, Appenzeller-Herzog C (2015). The antioxidant machinery of the endoplasmic reticulum: protection and signaling. Free Radic Biol Med..

[10] Wang J, Hu X, Jiang H (2016). ERS-PERK signaling pathway-mediated Nrf2/ARE-HO-1 axis: A novel therapeutic target for attenuating myocardial ischemia and reperfusion injury. Int J Cardiol..

[11] Zimmermann K (2015). Activated AMPK boosts the Nrf2/HO-1 signaling axis-A role for the unfolded protein response. Free Radic Biol Med..

[12] Zhu YF (2015). Allicin improves endoplasmic reticulum stress-related cognitive deficits via PERK/Nrf2 antioxidative signaling pathway. Eur J Pharmacol.

[13] Hetz CA (2007). ER stress signaling and the BCL-2 family of proteins: from adaptation to irreversible cellular damage. Antioxid Redox Signal..

[14] Oyadomari S, Mori M (2004). Roles of CHOP/GADD153 in endoplasmic reticulum stress. Cell Death Differ..

[15] Ye J (2014). L-carnitine attenuates H_2_O_2_-induced neuron apoptosis via inhibition of endoplasmic reticulum stress. Neurochem Int..

[16] Padilla A, Descorbeth M, Almeyda AL, Payne K, De Leon M (2011). Hyperglycemia magnifies Schwann cell dysfunction and cell death triggered by PA-induced lipotoxicity. Brain Res..

[17] Tomlinson DR, Gardiner NJ (2008). Glucose neurotoxicity. Nat Rev Neurosci..

[18] Eckersley L (2002). Role of the Schwann cell in diabetic neuropathy. Int Rev Neurobiol..

[19] Cinci L (2015). Oxidative, metabolic, and apoptotic responses of Schwann cells to high glucose levels. J Biochem Mol Toxicol..

[20] Lupachyk S, Watcho P, Obrosov AA, Stavniichuk R, Obrosova IG (2013). Endoplasmic reticulum stress contributes to prediabetic peripheral neuropathy. Exp Neurol..

[21] Lupachyk S, Watcho P, Stavniichuk R, Shevalye H, Obrosova IG (2013). Endoplasmic reticulum stress plays a key role in the pathogenesis of diabetic peripheral neuropathy. Diabetes..

[22] Gao Y (2013). Clinical research on patients of diabetic peripheral neuropathy treated with Tangluoning. China J Tradit Chin Med Pharm..

[23] Yang X (2015). Mechanism of Tang Luo Ning effect on attenuating of oxidative stress in sciatic nerve of STZ-induced diabetic rats. J Ethnopharmacol..

[24] Cullinan SB (2003). Nrf2 is a direct PERK substrate and effector of PERK-dependent cell survival. Mol. Cell. Biol..

[25] Freeman OJ (2016). Metabolic dysfunction is restricted to the sciatic nerve in experimental diabetic neuropathy. Diabetes..

[26] Mizisin AP (2014). Mechanisms of diabetic neuropathy: Schwann cells. Handb Clin Neurol..

[27] Liu J, Du L (2015). PERK pathway is involved in oxygen-glucose-serum deprivation-induced NF-κB activation via ROS generation in spinal cord astrocytes. Biochem Biophys Res Commun..

[28] Mota SI (2015). Oxidative stress involving changes in Nrf2 and ER stress in early stages of Alzheimer’s disease. Biochim Biophys Acta..

[29] Campana WM (2007). Schwann cells: activated peripheral glia and their role in neuropathic pain. Brain Behav Immun..

[30] Delaney CL, Russell JW, Cheng HL, Feldman EL (2001). Insulin-like growth factor-I and over-expression of Bcl-xL prevent glucose-mediated apoptosis in Schwann cells. J Neuropathol Exp Neurol..

[31] Askwith T, Zeng W, Eggo MC, Stevens MJ (2009). Oxidative stress and dysregulation of the taurine transporter in high-glucose-exposed human Schwann cells: implications for pathogenesis of diabetic neuropathy. Am J Physiol Endocrinol Metab..

[32] Yang X (2016). Paeoniflorin protects Schwann cells against high glucose induced oxidative injury by activating Nrf2/ARE pathway and inhibiting apoptosis. Journal of Ethnopharmacology..

[33] Rashid K, Sil PC (2015). Curcumin ameliorates testicular damage in diabetic rats by suppressing cellular stress-mediated mitochondria and endoplasmic reticulum-dependent apoptotic death. Biochim Biophys Acta..

[34] Kozlov AV (2009). Endotoxin causes functional endoplasmic reticulum failure, possibly mediated by mitochondria. Biochim Biophys Acta..

[35] Thon M, Hosoi T, Yoshii M, Ozawa K (2014). Leptin induced GRP78 expression through the PI3k-mtor pathway in neuronal cells. Sci Rep.

[36] Suyama K (2011). Overexpression of GRP78 protects glial cells from endoplasmic reticulum stress. Neurosci Lett.

[37] Cullinan SB, Diehl JA (2006). Coordination of ER and oxidative stress signaling: the PERK/Nrf2 signaling pathway. Int J Biochem Cell Biol..

[38] Bhakkiyalakshmi E, Sireesh D, Rajaguru P, Paulmurugan R, Ramkumar KM (2015). The emerging role of redox-sensitive Nrf2-Keap1 pathway in diabetes. Pharmacol Res..

[39] Palsamy P, Bidasee KR, Shinohara T (2014). Selenite cataracts: activation of endoplasmic reticulum stress and loss of Nrf2/Keap1-dependent stress protection. Biochim Biophys Acta.

[40] Wakabayashi N (2004). Protection against electrophile and oxidant stress by induction of the phase 2 response: fate of cysteines of the Keap1 sensor modified by inducers. Proc Natl Acad Sci USA.

[41] McCullough KD, Martindale JL, Klotz LO, Aw TY, Holbrook NJ (2001). Gadd153 sensitizes cells to endoplasmic reticulum stress by down-regulating Bcl2 and perturbing the cellular redox state. Mol Cell Biol.

[42] Gupta A (2015). PERK regulated mir-424(322)-503 cluster fine-tunes activation of ire1 and ATF6 during unfolded protein response. Sci Rep..

[43] Li X (2015). The role of HO-1 in protection against lead-induced neurotoxicity. Neurotoxicology..

[44] Logue SE, Cleary P, Saveljeva S, Samali A (2013). New directions in ER stress-induced cell death. Apoptosis..

[45] Vliet ARV, Verfaillie T, Agostinis P (2014). New functions of mitochondria associated membranes in cellular signaling. Biochim Biophys Acta..

[46] Sano R, Reed JC (2013). ER stress-induced cell death mechanisms. Biochim Biophys Acta.

[47] Dou G (2012). Deficiency of alphaB crystallin augments ER stress-induced apoptosis by enhancing mitochondrial dysfunction. Free Radic Biol Med..

[48] Daub, A., Sharma, P. & Finkbeiner, S. High-content screening of primary neurons: ready for prime time. *Curr Opin Neurobiol*. **19**, 537-543, 10.1016/j.conb.2009.10.002 19889533 (2009). 10.1016/j.conb.2009.10.002 PMC2787795 19889533

