# Supplementary material for: Tangluoning, a traditional Chinese medicine, attenuates in vivo and in vitro diabetic peripheral neuropathy through modulation of PERK/Nrf2 pathway
Source: Sci Rep. 2017 Apr 21;7:1014. doi: 10.1038/s41598-017-00936-9 (PMC5430716; doi:10.1038/s41598-017-00936-9)
Supplement: Supplementary file 1 — Dataset 1 [file 41598_2017_936_MOESM1_ESM.zip]

**
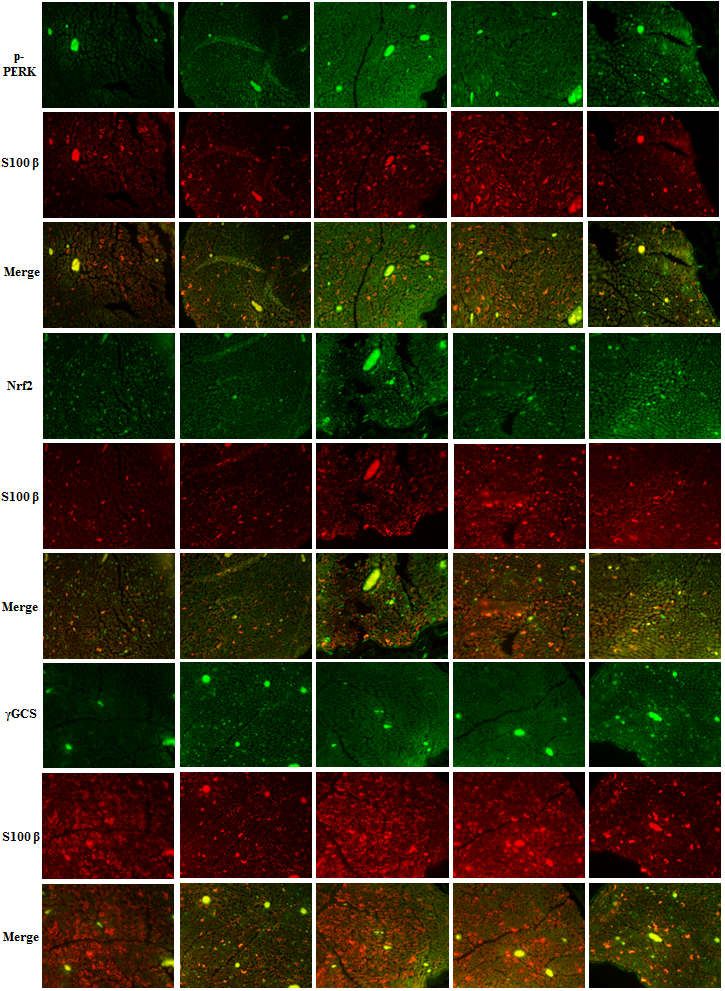
**

**Suppl. Figure 1.** Immunofluorescence images showing double immunostaining of p-PERK, Nrf2 and γGCS located in Schwann cells in sciatic nerve sections (40× magnification). FITC-conjugated affinipure goat anti-rabbit as secondary antibody (green) were used to stain p-PERK, Nrf2 and γGCS. TRITC-conjugated affinipure goat anti-mouse as secondary antibody (red) was used to stain S-100 β. White arrows shows positive staining.

**Suppl. Table 1. The formula of Tangluoning per dose**

| Chinese name | Plant source | Medicinal parts | Amount in preparation (g) |
| --- | --- | --- | --- |
| Huangqi | *Astragalus membranaceus* (Fisch) Bge. | radix | 15 |
| Danshen | *Salvia Miltiorrhiza* Bge. | radix | 15 |
| Jixueteng | *Spatholobus suberectus* Dunn | caulis | 15 |
| Mugua | *Chaenomeles speciosa* (Sweet) Nakai | fruit | 15 |
| Gouji | *Cibotium Barometz* (L.) J. Sm. | rhizome | 15 |
| Niuxi | *Achyranthes bidentata* Bl. | radix | 12 |
| Chishao | *Paeonia lactiflora* Pall. | radix | 12 |
| Yanhusuo | *Corydalis yanhuasuo* W. T. Wang | rhizome | 10 |

**Suppl. Table 2. The formula of Tangluoning per dose**

| Sample | Linearity | | |
| --- | --- | --- | --- |
|  | Equation | R^2^ | Range  (ng/mL) |
|  |  |  |  |
| chlorogenic | y=0.00003x+0.00214 | 0.9999 | 6.0-387.0 |
| albiflorin | y=0.00012x+0.00058 | 0.9999 | 12.5-800.0 |
| paeoniflorin | y=0.00008x+0.01124 | 0.9995 | 15.0-980.0 |
| rosmarinic acid | y=0.00004x+0.02346 | 0.9995 | 23.1-740.0 |
| salvianolic acid B | y=0.00010x-0.00215 | 0.9999 | 15.9-1022.0 |

Five major components (chlorogenic, albiflorin, paeoniflorin, rosmarinic acid and salvianolic acid B) were clearly detected with baseline separation. The content of chlorogenic, albiflorin, paeoniflorin, rosmarinic acid and salvianolic acid B in TLN extract was 5.368, 9.779, 76.786, 8.116 and 99.013 mg/g respectively

**Suppl. Table 3. List of antibodies used.**

| **Antigen** | **Type** | **Provider** | **Application** | **Dilution** |
| --- | --- | --- | --- | --- |
| Nrf2 | Rabbit polyclonal No. sc-722 | Santa Cruz | IHC  HCA | 1:50  1:100 |
| γGCSc | Rabbit polyclonal  No. sc-22755 | Santa Cruz | IHC  HCA | 1:50  1:100 |
| PERK  (phospho T980) | Rabbit polyclonal  No. ab156919 | Abcam | IHC  HCA | 1:100  1:200 |
| S-100 β | Mouse monoclonal  No. 393919 | Santa Cruz | IHC | 1:100 |
| GRP78 | Mouse monoclonal  No. sc-376768 | Santa Cruz | WB | 1:1000 |
| Keap1 | Rabbit polyclonal  No. sc-33569 | Santa Cruz | WB | 1:2000 |
| GADD153 | Mouse monoclonal  No. sc-7351 | Santa Cruz | WB | 1:1000 |
| Bcl-2 | Rabbit polyclonal  No. sc-492 | Santa Cruz | WB | 1:1000 |
| Bax | Mouse monoclonal  No. sc-7480 | Santa Cruz | WB | 1:1000 |
| Caspase-3 | Rabbit polyclonal  No. sc-7148 | Santa Cruz | WB | 1:1000 |
|  |  |  |  |  |
| HO-1 | Rabbit monoclonal  No. ab68477 | Abcam | WB | 1:10000 |


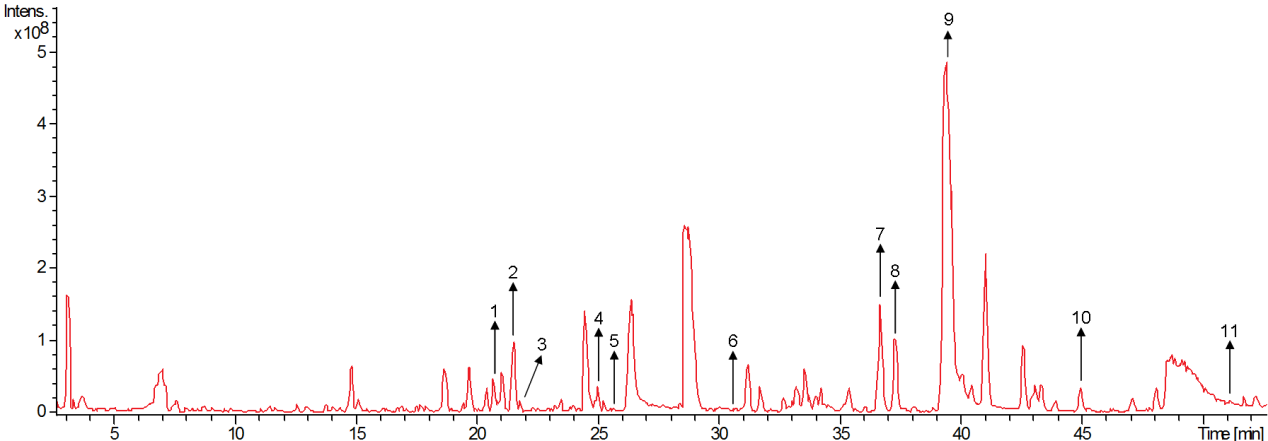


**Suppl. Figure 2. HPLC-MS/MS total ion chromatogram (TIC) in negative ion mode of extract of TLN**. In a previous study, eleven compounds have been identified in TLN according to the HPLC-MS/MS. (Stationary phase: Alltima C18 column (250 mm × 4.6 mm, 5 μm), flow rate: 1 mL/min). (Each compound indicated by peak number: 1. oxypaeoniflora, 2. chlorogenic acid, 3. albiflorin, 4. paeoniflorin, 5. ginkgolide C, 6. ellagic acid, 7. rosmarinic acid, 8. salvianolic acid A, 9. salvianolic acid B, 10. calycosin, 11. astragaloside A) [suppl. Reference 1]

**Suppl. Reference 1**

X. Yang, W. Yao, Q. Li, H. Liu, H. Shi, Y. Gao, L. Xu. Mechanism of Tang Luo Ning effect on attenuating of oxidative stress in sciatic nerve of STZ-induced diabetic rats. J. Ethnopharmacol, 2015; 174: 1-10.
